# Dysarthria-Clumsy Hand Syndrome in a Patient with a Caudate Nucleus Stroke: A Case Report

**DOI:** 10.5811/cpcem.46593

**Published:** 2025-10-02

**Authors:** Janan Niknam, Sarah Al-Zaher, Sivarma K. Kotikalapudi

**Affiliations:** * William Carey University College of Osteopathic Medicine, Hattiesburg, Mississippi; † Southern Star Medical Group, Hattiesburg, Mississippi

**Keywords:** *dysarthria-clumsy hand syndrome*, *caudate nucleus*, *lacunar stroke*, *hypertension*, *case report*

## Abstract

**Introduction:**

Dysarthria-clumsy hand syndrome (DCHS) is a rare finding reported in lacunar strokes. Lesions in various anatomic locations have been reported. While the association of DCHS with a caudate nucleus lesion has been documented, such reports remain infrequent.

**Case Report:**

In this case we present a 52-year-old male who presented with DCHS following a stroke affecting the caudate nucleus. Neurological examination revealed left-sided motor deficits. Magnetic resonance imaging confirmed an isolated infarct in the right caudate nucleus.

**Conclusion:**

This case report describes a patient with dysarthria-clumsy hand syndrome, due to a lesion in the caudate nucleus and the internal capsule.

## INTRODUCTION

Stroke has been one of the leading causes of disability in the United States in recent years.^1^ Among the two major subtypes of stroke, ischemic and hemorrhagic stroke, ischemic strokes are much more commonly seen. Lacunar strokes are a somewhat rare subtype of ischemic strokes, accounting for 20–30% of cases.^2^ It has previously been shown that most cases of lacunar strokes are caused by occlusive lesions affecting small, perforating branches of large cerebral arteries that supply deep subcortical structures in the brain.^3^ The pathology has been described as fibrinoid necrosis of the vessel walls, as well as segmental arteriolar disorganization, happening as a result of uncontrolled hypertension.^3^ Therefore, although traditional risk factors for stroke can predispose individuals to lacunar strokes, hypertension has been found to be a more powerful risk factor for lacunar strokes compared to other types of ischemic strokes.^4^

Multiple clinical syndromes have been described in patients as a result of lacunar strokes. These syndromes include pure motor hemiparesis, ataxic hemiparesis, pure sensory stroke, sensorimotor stroke, and dysarthria-clumsy hand syndrome (DCHS).^5^ Among these, DCHS is the least common and poorly studied syndrome. Typical symptoms observed in DCHS include unilateral facial weakness, moderate dysarthria, slight weakness observed in the arms and legs, and slowing of rapid repetitive movement of the hand and foot.^6^ Dysarthria-clumsy hand syndrome is an exceedingly rare syndrome, accounting for only 4.6% of 283 possible lacunar strokes and 7.1% of 210 probable lacunar strokes in the North American Symptomatic Carotid Endarterectomy Trial.^7^ We present the case of a 52-year-old male presenting with DCHS following a lacunar stroke affecting the caudate nucleus.

## CASE REPORT

A 52-year-old, right-handed male truck driver with a past medical history of poorly controlled hypertension and type II diabetes mellitus presented to the emergency department with new-onset slurred speech, left facial droop, and diaphoresis. He denied dizziness, dysphagia, vision changes, and palpitations. The patient’s last known normal at 5:15 am, making the total symptom duration at least 10 hours and 10 minutes. Upon examination, the patient was alert and oriented. On admission, his vital signs were as follows: temperature, 98.6 °F; heart rate, 88 beats per minute; respiratory rate, 19 breaths per minute; blood pressure, 174/88 millimeters of mercury; and oxygen saturation, 98% on room air. His physical exam confirmed a left-sided facial droop and slurred speech. The physical exam also showed motor weakness of 4/5 in the left upper and lower extremities. The remainder of the physical exam was unremarkable. His National Institute of Health Stroke Scale (NIHSS) score was determined to be two.

On admission, the patient’s hemoglobin A1c was measured at 12% (reference range: 4–5.6%), further indicating inadequate control of his diabetes mellitus. A complete metabolic panel indicated a potassium level of 3.0 millimoles per liter (3.5–5 mmol/L), thought to be partly due to his use of lisinopril. The remainder of his laboratory results, including his estimated glomerular filtration rate, blood urea nitrogen, and creatinine were unremarkable. A computed tomography with angiography (CTA) of the neck showed no obvious proximal stenosis; however, the reported findings were limited due to motion artifact and contrast bolus timing issues. A CT of the head showed evidence of subacute ischemia in the white matter adjacent to the caudate nucleus on the right. At the time of presentation, the therapeutic window for thrombolytic therapy with tenecteplase had passed. The patient’s home dose of lisinopril was held while at the hospital to allow permissive hypertension.

The patient was admitted for stroke. The workup included a carotid Doppler ultrasound, a transthoracic echocardiogram (TTE), and a magnetic resonance imaging (MRI) of the brain without contrast (Image). The carotid Doppler ultrasound revealed no limiting lesions in the right or left carotid arteries. Similarly, the TTE revealed no abnormalities in the patient’s heart. The brain MRI indicated a lesion in the right caudate nucleus, extending into the posterior limb of the internal capsule on the right.

*CPC-EM Capsule*

What do we already know about this clinical entity?

*Dysarthria-clumsy hand syndrome (DCHS) is a rare lacunar stroke syndrome presenting with unilateral facial weakness, dysarthria, and motor weakness. It is one of the least common syndromes*.

What makes this presentation of disease reportable?

*This case reports DCHS from a lacunar infarct involving both the caudate nucleus and posterior limb of the internal capsule, a presentation not commonly documented*.

What is the major learning point?

*The major learning point is that DCHS can result from lacunar infarcts involving both the caudate nucleus and internal capsule*.

How might this improve emergency medicine practice?

*This case can help emergency medicine physicians consider DCHS as a possible diagnosis in a patient presenting with a lacunar stroke affecting the caudate nucleus*.

The patient’s slurred speech had resolved spontaneously by his discharge date, two days after admission. Some presenting symptoms, however, were found to persist. These symptoms included a decreased nasolabial fold on the left side, deviation of the tongue and the uvula to the right side, and left-sided motor deficits. The patient was started on atorvastatin as well as dual antiplatelet therapy, consisting of aspirin 81 mg and clopidogrel 75 mg. He was instructed to continue the dual antiplatelet therapy for 21 days.

The patient was seen two weeks later for a follow-up internal medicine appointment. He complained of difficulty maintaining focus while at work and diaphoresis. He also complained of dysarthria, possibly due to the persistence of his left-sided facial droop and weakness of the left hand. Physical exam indicated right tongue and uvula deviation, as well as decreased strength in the left hand. Motor strength was 4/5 in the left upper extremity, unchanged compared to strength upon discharge from the hospital; the left lower extremity strength, however, was 5/5. The remainder of the physical exam yielded no significant findings. During this appointment, the patient provided informed consent for the use of his medical information in the writing of this case report.

## DISCUSSION

We describe a case of DCHS following a stroke in the caudate nucleus, with the lesion extending into the posterior limb of the internal capsule in a patient with poorly controlled hypertension and diabetes mellitus. Dysarthria-clumsy hand syndrome is the most infrequent syndrome caused by a lacunar stroke. The syndrome can consist of dysarthria and dysphagia as well as weakness of one hand. A range of lesions have been previously associated with DCHS. One study showed that most patients with DCHS have pontine infarctions.^8^ In contrast, a more recent study implicated lesions in the anterior limb of the internal capsule in the development of DCHS.^9^ Wouter et al (1999) reported two patients with DCHS, with one patient having a lesion in the putamen and the other in the caudate nucleus. Both patients in that study also exhibited involvement of white matter tracts.^10^ Our patient reported symptoms and observable signs aligning with previous descriptions of DCHS. However, the clinical presentation observed in the patient, in conjunction with the involvement of the caudate and the posterior limb of the internal capsule, offers valuable clinical insight into the course and spectrum of manifestation of DCHS in the literature.

The caudate nucleus, a part of the basal ganglia, is a potentially vulnerable region to ischemia and damage secondary to disconnection due to damage to white matter following a cerebrovascular accident.^11^. Various consequences of caudate nucleus strokes have been described in the literature. For instance, subacute strokes in the caudate region have been shown to be associated with preservation, independent of the presence of hemi-neglect.^12^ Lesions involving the caudate have also been shown to be associated with post-stroke dysphagia.^13^ Studies have also shown lesions involving the posterior limb of the internal capsule to be correlated with more severe motor deficits after a stroke.^14^ The present case demonstrated a lacunar infarction involving the caudate nucleus, extending into the posterior limb of the internal capsule.

## CONCLUSION

Lacunar infarcts can present with a variety of symptoms that can be diverse and subtle at times. The present report showcases a rare presentation associated with lacunar infarcts due to a lesion in the caudate nucleus and the internal capsule. This report presents important insights for the management and prognosis of patients with lacunar infarcts affecting these anatomical regions.

## Figures and Tables

**Image:**
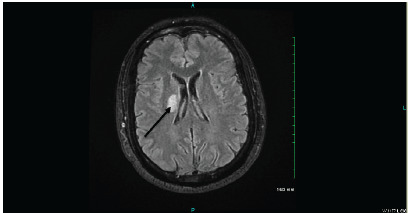
Axial T2-weighted magnetic resonance imaging (MRI) image showing hyperintensity (arrow) in the right caudate nucleus, consistent with an acute ischemic stroke.

## References

[1] Murray CJL, Lopez AD (2013). Measuring the global burden of disease. N Engl J Med.

[2] Caplan LR (2015). Lacunar infarction and small vessel disease: pathology and pathophysiology. J Stroke.

[3] Fisher CM (1968). The arterial lesions underlying lacunes. Acta Neuropathol.

[4] You R, McNeil JJ, O’Malley HM (1995). Risk factors for lacunar infarction syndromes. Neurology.

[5] Donnan GA, Norrving B (2009). Lacunes and lacunar syndromes. Handb Clin Neurol.

[6] Fisher CM (1967). A lacunar stroke. The dysarthria-clumsy hand syndrome. Neurology.

[7] Inzitari D, Eliasziw M, Sharpe BL (2000). Risk factors and outcome of patients with carotid artery stenosis presenting with lacunar stroke. North American Symptomatic Carotid Endarterectomy Trial Group. Neurology.

[8] Glass JD, Levey AI, Rothstein JD (1990). The dysarthria-clumsy hand syndrome: a distinct clinical entity related to pontine infarction. Ann Neurol.

[9] Arboix A, Bell Y, García-Eroles L (2004). Clinical study of 35 patients with dysarthria-clumsy hand syndrome. J Neurol Neurosurg Psychiatry.

[10] Schonewille WJ, Tuhrim S, Singer MB (1999). Diffusion-weighted MRI in acute lacunar syndromes. Stroke.

[11] Looi JCL, Tatham V, Kumar R (2009). Caudate nucleus volumes in stroke and vascular dementia. Psychiatry Res Neuroimaging.

[12] Nys GMS, van Zandvoort MJE, van der Worp HB (2006). Neuropsychological and neuroanatomical correlates of perseverative responses in subacute stroke. Brain.

[13] Im I, Jun JP, Hwang S (2018). Swallowing outcomes in patients with subcortical stroke associated with lesions of the caudate nucleus and insula. J Int Med Res.

[14] Puig J, Pedraza S, Blasco G (2011). Acute damage to the posterior limb of the internal capsule on diffusion tensor tractography as an early imaging predictor of motor outcome after stroke. AJNR Am J Neuroradiol.

